# Improving the Health Literacy of Patients: A Qualitative Survey of Student Pharmacists

**DOI:** 10.7759/cureus.34716

**Published:** 2023-02-07

**Authors:** Sarah John, Mark S Abdelmalek, Justina Refela, Frank Breve

**Affiliations:** 1 Department of Pharmacy, Temple University, Philadelphia, USA

**Keywords:** health literacy, patient counseling, patient education, pharmacy, student pharmacist

## Abstract

Background

Patients interacting with the multiple moving parts of the healthcare system may not fully understand all the information provided to them. They find themselves misinformed or unaware of certain facts pertaining to their health. Community pharmacists, who are readily accessible, are occupationally situated in such a way that makes them the most ideal candidates to impact and improve patients’ health literacy.

Aims and objectives

The aim of this research is to identify and examine how pharmacists can aid their patients and help them toward adequate health literacy.

Methods

First-year pharmacy candidates enrolled in the Doctor of Pharmacy (PharmD) program at Temple University School of Pharmacy were given an optional, essay-based assignment titled, “How can pharmacists improve the health literacy of the patients they serve?” Students were given one week to respond to the prompt, and those who completed the assignment were awarded bonus points toward the final examination. Of the 145 students given the potential to complete the assignment, only 100 students participated in the assignment. These essays were subsequently read, categorized, and analyzed.

Results

The essays were categorized into six main perspectives: educating patients, using simple language, providing resources, creating a trusting patient-pharmacist relationship, sending medication reminders, and providing translation services. More than 30% of the student responses emphasized that educating patients about their medication side effects and disease states will help them better understand their medical needs. About another 30% of the students suggested that using simple, everyday language and providing translators, when necessary, will help patients deal with their health problems. The remaining suggested either creating a safe relationship with patients, providing reliable resources such as pamphlets and visual aids, or sending patient-specific reminders.

Conclusion

Although these suggestions are not new to the world of pharmacy and have been around before, the difficulty lies in practical application in a world that seems busier now than ever before. However, within the six student perspective response categories, most can be incorporated into one patient interaction. By creating a trusting relationship with the patient and counseling them while using simple language and translators, if necessary, teaching them about their medications, and providing the necessary outside resources, pharmacists can improve the health literacy of their patients.

## Introduction

Health literacy is defined as “the degree to which individuals have the capacity to obtain, process, and understand basic health information and services needed to make appropriate health decisions” according to the Institute of Medicine and the National Library of Medicine [1]. In the most updated study, which was from 2003, the results from the National Assessment for Adult Literacy showed that only 12% of US adults had proficient health literacy [2]. Within the same study, race/ethnicity was found to be an additional factor in health literacy with Hispanic/Black adults recording the lowest health literacy scores, while White/Asian adults recording the highest health literacy scores [2]. In 1997, the World Health Organization (WHO) added the role of teacher to its preexisting six defined roles of a pharmacist to create a new model called the “seven-star pharmacist” [3]. Counseling, which encompasses teaching, listening, and building a trusting patient relationship, when intertwined with the use of simple language, translators (if necessary), and external resources, gives patients all the necessary tools they need to build their bridge of knowledge and overcome the health literacy gap that exists between them and pharmacists.

According to a Centers for Disease Control and Prevention (CDC) review titled, “Community Pharmacy and Medication Therapy Management,” the Ohio-based study found that control of hypertension increased by approximately 70% in their 5,000 patient population who received medication therapy management (MTM) from a pharmacist [4]. MTM is an effective form of patient education. Medication therapy management (MTM) is a service or group of services that optimize the therapeutic outcomes for individual patients [4]. A core component of MTM is medication therapy review (MTR). The goal of this component is to address any of the patients’ concerns or problems, improve the patient’s knowledge of medications, and encourage patients to self-manage their medications and health conditions [4]. MTM interventions include providing verbal education and training to enhance the patient’s understanding of their medications, and it helps provide resources to help patients adhere to their medications [4].

Without the necessary comprehension of health information, it is difficult to make health-related decisions. To help shed light on this issue, pharmacy students were asked to delineate their opinions and ideas on the role of pharmacists in the education of patients and their impact on health literacy. Using essays, pharmacy students shared their insights on how they can, as healthcare providers, best serve their communities and patients. The students also provided specific insight on how to most effectively perform MTM. This assignment allowed them to express their ideas and what they could bring to the conversation as valuable members of healthcare teams. This compilation puts into words their opinions as student pharmacists, as well as what they have seen done in their places of work.

The importance of this study is that it offers a different perspective from the lens of future pharmacists. These student pharmacists, having only completed one semester at the time of the essay assignment, could be more effectively viewed as a pool of patients rather than students. In this light, these perspectives on how to improve patients’ health literacy are valued in that they look at the issue from multiple angles, including both the individual students and the experiences of their families, into one formally presentable study. Therefore, this offers the pharmacy community an insight into how patients and their families are truly feeling and hoping to be served.

## Materials and methods

First-year pharmacy candidates enrolled in the Doctor of Pharmacy (PharmD) program at Temple University School of Pharmacy were required to take a healthcare course. In this class, Professor Breve offered them the option of completing an extra-credit assignment of writing a short essay about, “What can pharmacists do to improve the health literacy of the patients they serve?” This question was based on the professor’s teaching and work experience with his students. The instructor made it clear to the class that the comments and answers from this optional assignment might appear in a future publication. There were no multiple-choice options or suggestions, and no word length requirements, allowing for the maximum range of inputs and novel ideas. Students had about half of the semester to submit the assignment.

When all essays had been turned in, two student investigators (Sarah John and Mark S. Abdelmalek) were to tabulate the student responses, divide the essays between them, and then describe the predominant theme in each essay in one word or a short phrase. Since this was a “free form” assignment, there was no preset list of categories, but rather, the categories were to be derived from the essays. Upon the completion of the analysis of the qualitative data, the lists of categories were compared and synthesized. Notable remarks and quotations were highlighted in each essay. Both investigators reviewed a portion of the essays originally analyzed by the other, and there was agreement between the investigators.

## Results

This optional assignment was offered to 145 students, of whom 100 turned in a completed essay for analysis, providing a response rate of 68.9%. Forty-five students opted to not participate in this assignment. Two students volunteered to analyze the results, each of whom assessed 50 manuscripts and then consolidated their findings. All papers could be assigned to one of the six available categories (Table 1). Within each perspective, recurrent themes were noted for discussion.

**Table 1:** Six Main Student Perspectives on Improving Health Literacy of Patients *Patient reminders (text messages and phone calls), hosting public healthcare seminars, and pharmacy appointments

Educating Patients	Using Simple Language	Providing Resources	Creating a Relationship	Other*	Providing Translators/Interpreters
35	26	16	10	7	6

Out of the 100 students, 35 suggested pharmacists ought to spend the time and teach patients about their medications. Patient education is much more extensive than instructing them to take one tablet by mouth daily. Rather, it involves explaining the disease state, describing how the medication functions, and clarifying common side effects.

Twenty-six students recommended using simple language. As future healthcare professionals, student pharmacists are constantly hearing and learning new medical terminology, whereas for a majority of patients, the pharmacy counter may be their first encounter with terms such as hypertension or syncope. By using simple, everyday words and phrases to explain disease states, medication mechanisms of action, or common side effects, patients may gain a better understanding of their condition and treatment and become more engaged.

Sixteen students said that providing resources would help patients better understand the medications they are taking. Resources that were mentioned included pamphlets and brochures that summarize the drugs’ key counseling points, video links that provide more information for the patient, and, most importantly, reliable websites where patients can do further research. It is difficult to grasp all the information being presented at the pharmacy counter, and thus, these resources are essential for further clarification and future patient reference.

Ten students mentioned creating a relationship between the patient and the pharmacist. This vital relationship can only be built when pharmacists go out of their way to prove that they are there to care and advocate for patients, and have their best interests in mind. If the patients do not trust the healthcare providers, or if the healthcare providers do not spend adequate time with their patients, then they will not go to them for questions, causing them to turn to unreliable sources for their health-related problems.

Seven students discussed other options to help with patients’ health literacy. Of those, a few students mentioned sending text messages or initiating phone calls to patients as reminders about their medications, notifying them when their medications are ready to be picked up. Others mentioned hosting a public healthcare seminar to educate patients about basic medical terminology and disease states, allowing for further education and beneficial question and answer (Q&A) with healthcare specialties of such disease states.

Six students recommended having readily accessible interpreter hotlines or mobile translation services to better serve their patients. Up to 20% of Americans speak a language other than English at home, including many who were born in this country [5]. Interpreters help bridge the gap by serving as facilitators to convey information. Talking to patients in their native language allows them to better understand and engage in their own health journey.

## Discussion

According to the 2003 National Assessment of Adult Literacy, 14% of adults are below basic for health literacy, and it showed that White and Asian/Pacific Islander adults have higher health literacy scores compared to other races [2]. In a study held at a primary care clinic in Shreveport, Louisiana, approximately one-third of their patients were reading at or below the sixth-grade level [6]. People with low health literacy are more likely to make medication errors, have poor health outcomes, and have trouble managing chronic diseases, such as diabetes and hypertension [7]. These student essays described from different perspectives how healthcare professionals can improve the health literacy of our patients.

Within the first perspective, the most recurring theme in these essays was simply teaching patients, in simple layman’s terms, how the medication they are taking works and what adverse effects have been reported. Using techniques such as the teach-back method can help patients be more actively involved in their health plans [8]. During counseling sessions, asking open-ended questions and speaking more slowly and clearly during instruction can help patients understand. Pharmacists should talk to patients in a calming manner and be willing to answer all their questions. Pharmacists should also confirm with the patient what was written on the medication label and not just assume that because it is written that the patient understands what it means. This can ensure that the patient knows which medication to take, how to take it, and its purpose. By explaining, for example, to a patient with hypertension that their “water pill” will cause them to urinate more frequently, taking their medication in the morning would make more sense. However, if patients are only instructed to take their medication in the morning without further explanation, they may forget one morning, remember, and take it at night, and then complain of nocturia.

Another example is that, if the patient is a first-time inhaler user, the pharmacist should show the patient how to use the inhaler, the type of breath to take, where to store it, etc. Then, the patient should teach back what the pharmacist taught them. This method allows the patients to perform a task or correctly use a device in front of the patients, and the pharmacist can confirm if they are using it properly. Pharmacists can also follow the “Ask Me 3” campaign, which consists of three important questions: what is my main problem, what do I need to do, and why is it important for me to do this [9]? This will help patients have a better understanding of their prescriptions and ensure medication safety. The more patients understand why their medications are important, the more they will be willing to take the necessary steps toward improving their health. Therefore, teaching patients about their medications and utilizing the teach-back method assesses their understanding and aids in improving patient health literacy.

Alongside teaching, it is important for pharmacists to correct misconceptions that patients may have heard, including the popular idea that there is a difference in efficacy between brand name and generic medications. Unfortunately, as one student put it, “(patients) assume generic drugs are like generic cereal” and that generic drugs are basically a “knockoff” or cheap imitation of the real product. This error has become very prevalent to the point where we see patients willing to pay extra out-of-pocket costs to get the “better” brand-name product. Therefore, students have suggested simply explaining that brand and generic products have the same active pharmaceutical ingredients and work in the same exact way. Perhaps by explaining to patients that Tylenol® is the exact same drug as acetaminophen, patients can be made aware of cost-saving options.

More than 25% of the students stated that using simple language can help more patients understand their health. Better health information may help patients make better health decisions. Writing medical information in everyday language is important for patients to understand. Doctors may provide too much information at once for the patient to take in. As pharmacists, we also need to work on effective communication strategies such as speaking slowly, defining technical and medical terms, and constantly asking questions. Medical terminology can be a communication barrier for some patients. Another approach is to avoid abbreviations and use more specific directions on the medication labels. Instead of writing “po,” one should write “by mouth.” Instead of writing “take after you eat,” one should write “take one tablet (25 mg) after you eat breakfast every day.” The latter is more specific and helps the patient understand how much medication to take and when to take it. Drug labels should have the potential to create a patient-centered system of care that will help patients take medications more safely. Another method to help patients is to provide mandatory literacy training for technicians. Technicians usually spend a lot of time helping patients and trying to answer all their health questions. If they are trained in medical terminology, they can help patients with their medication understanding.

Another avenue to improving patient health literacy is by utilizing a streamlined technology platform containing all patient-specific medication information [10]. Although not commonly seen in the community setting, a patient portal that shows a clear list of medications, along with route, dose, frequency, side effects, and, very importantly, an image of the drug itself for reference, would greatly benefit patients. The simple image feature will allow patients to better put “name to the face” so to speak rather than having them refer to their medication as “the blue water pill” or “the green blood pressure drug.” Always having access to their list of medications via an encrypted smartphone application will allow for better, safer quality of care in the instance of an emergency room visit or hospitalization.

Perhaps one of the greatest means of increasing patients’ health literacy is by providing reliable resources (from audiovisual supplements to research articles) for patients to understand their medications and health conditions. Visuals, such as pictures and diagrams, make complicated information easier to understand [11]. Some students, however, pointed out that patients may doubt or question the information they receive because they read something different online or heard otherwise. In light of the pandemic, information was not readily available, but at a time when people did not know what organizations they could trust, pharmacists emerged as a trusted resource, and so, it is those same pharmacists who have the reach and ability to educate patients. Additional resources include pharmacy phone lines and online chat features, which allow patients to communicate with healthcare providers in real time and ask all their questions in the comfort of their homes. Patients should be encouraged to do their own research online being guided to reputable medical websites.

One major step toward patient safety and increasing health literacy is to create a trusting relationship between the pharmacist and the patient. Trust is a key component in creating a safe relationship between the patient and the pharmacist. This relationship allows patients to safely ask questions about their health without judgment or embarrassment. Unfortunately, as one essay also pointed out, there is a large stigma that pharmacists take financial advantage of their patients, either by raising prescription prices or dispensing “costly” brand-name drugs as opposed to their “cheaper” generic bioequivalent counterparts. This notion can fracture the already frail and brittle trust established between patients and healthcare providers. Therefore, few students have stressed the importance of the role that pharmacists can play in helping patients navigate the complicated world of drug prices.

One of the other suggestions to improve health literacy was by sending text message refill reminders. These automated reminders prompt the patient to refill their prescriptions through the phone [12]. It can also serve as a quick medication overview and an aid to medication adherence. These text message reminders should also include the name, dose, frequency, and indication of their medication, as well as an image of what it looks like. By sending these reminders, patients can always go back to these messages and remember all the key points of their medication. Another mentioned providing yearly health seminars for the public to better understand different types of medications and disease states. Having a health fair with different booths allows patients to go to the booth that applies to them. For example, if they go to the diabetes booth, they can learn more about the causes of diabetes, its consequences, and different types of medications, and interact with experts in that area.

Language barriers can be a major issue in the medical world. When patients do not understand the healthcare professions or speak the same language, the quality of care can be lowered [13]. Also, when there is a language barrier issue, it can impede the patient’s understanding of their medical condition or medications. Providing translated medication guides for patients to read and understand, and a translation hotline available for patients to ask any questions can be beneficial to the patient. As for interpreter systems, they may require an additional cost to patients, but providing them free of charge would remove that barrier in the outpatient setting. The use of a 24/7 translation line can be used by healthcare providers to help patients. For immediate use, pharmacists can also utilize online translation sites, such as Google Translate, for immediate translation of simple phrases.

This study has some limitations and strengths. The limitation is that this study was only qualitative, and it only depended on a sample of first-year pharmacy students. The strength is that the students responded in the form of an essay and were not limited to multiple-choice selections. By using an essay format, analysis from each pharmacy student’s unique perspective was compiled together to create a clear picture of how pharmacists can improve the overall health literacy of their patients.

## Conclusions

Pharmacists are vital healthcare professionals in the medical field. As pharmacists, we can help improve patients’ health literacy and be their guide in a confusing world of medical terminology. We must take into consideration offering realistic and attainable practices that will not further strain our teams while still providing our patients with the care in which they entrust to us. Within the six student perspective response categories, almost all of them can be incorporated into one simple interaction without it taking too much time. Based on this research study, it was found that by creating a trusting relationship with the patient and counseling them while using simple language and translators, teaching them about their medications, and providing the necessary outside resources, pharmacists can improve the health literacy of their patients.
