# Correction: Improving the Health Literacy of Patients: A Qualitative Survey of Student Pharmacists

**DOI:** 10.7759/cureus.c267

**Published:** 2025-08-26

**Authors:** Sarah John, Mark S Abdelmalek, Justina Refela, Frank Breve

**Affiliations:** 1 Department of Pharmacy, Temple University, Philadelphia, USA

The Ethics Statement and Conflict of Interest Disclosures section of this article has been corrected to indicate that this study did involve human subjects.

